# Is it the coracobrachialis superior muscle, or is it an unidentified rare variant of coracobrachialis muscle?

**DOI:** 10.1007/s00276-021-02773-y

**Published:** 2021-05-26

**Authors:** Łukasz Olewnik, Nicol Zielinska, Łukasz Gołek, Paloma Aragonés, Jose Ramon Sanudo

**Affiliations:** 1 Department of Anatomical Dissection and Donation, Medical University of Lodz, Lodz, Poland; 2 Department of Orthopedics Surgery. Hospital Santa Cristina, Madrid, Spain; 3 Department of Human Anatomy and Embryology, Facultad de Medicina, Universidad Complutense de Madrid, Madrid, Spain

**Keywords:** Anatomical variations, Coracobrachialis muscle, Coracobrachialis longus, Coracobrachialis brevis, Coracobrachialis superior

## Abstract

The coracobrachialis muscle (CBM) originates from the apex of the coracoid process, in common with the short head of the biceps brachii muscle, and from the intermuscular septum. The CBM demonstrates variability in both the proximal and distal attachment, with some extremely rare varieties, such as the coracobrachialis superior, coracobrachialis longus and coracocapsularis muscle. This case report describes an extremely rare variant of the coracobrachialis superior muscle, or a very rare variant of the CBM. Our findings highlight the importance of muscle variants in the shoulder region, especially the coracoid region, and are significant for radiologists, anatomists, physiotherapists and surgeons specializing in the shoulder joint.

## Introduction

Until recently, it was believed that the most morphologically variable muscles on the upper limb were the biceps brachii and palmaris longus; however, the latest research indicates that they have been joined by the coracobrachialis muscle (CBM) [16, 20]. The CBM belongs to the anterior group of the arm, together with the biceps brachii and brachialis muscle; however, it is smaller.

The CBM has significant variability with regard to both the proximal and distal attachment. Interestingly, recent studies have also highlighted its variation regarding the occurrence of additional muscle bellies or muscle belly heads. Morphological variations have also been noted between the CBM and the musculocutaneous nerve, namely, the nerve does not pierce the muscle. Recent case reports indicate the presence of a coracobrachialis longus muscle (CBL), or an atypical relationship between the branches of the brachial plexus and CBM. In addition, some extremely rare varieties have also been mentioned, such as the coracobrachialis superior (CBS) and coracocapsularis muscle.

This case report describes an extremely rare variant of the CBS or very rare variant of the CBM.

## Case report

The right upper limb from a female cadaver that was 78-years old at death underwent routine anatomical dissection for research and teaching purposes at the University Complutense of Madrid.

**Fig. 1:**
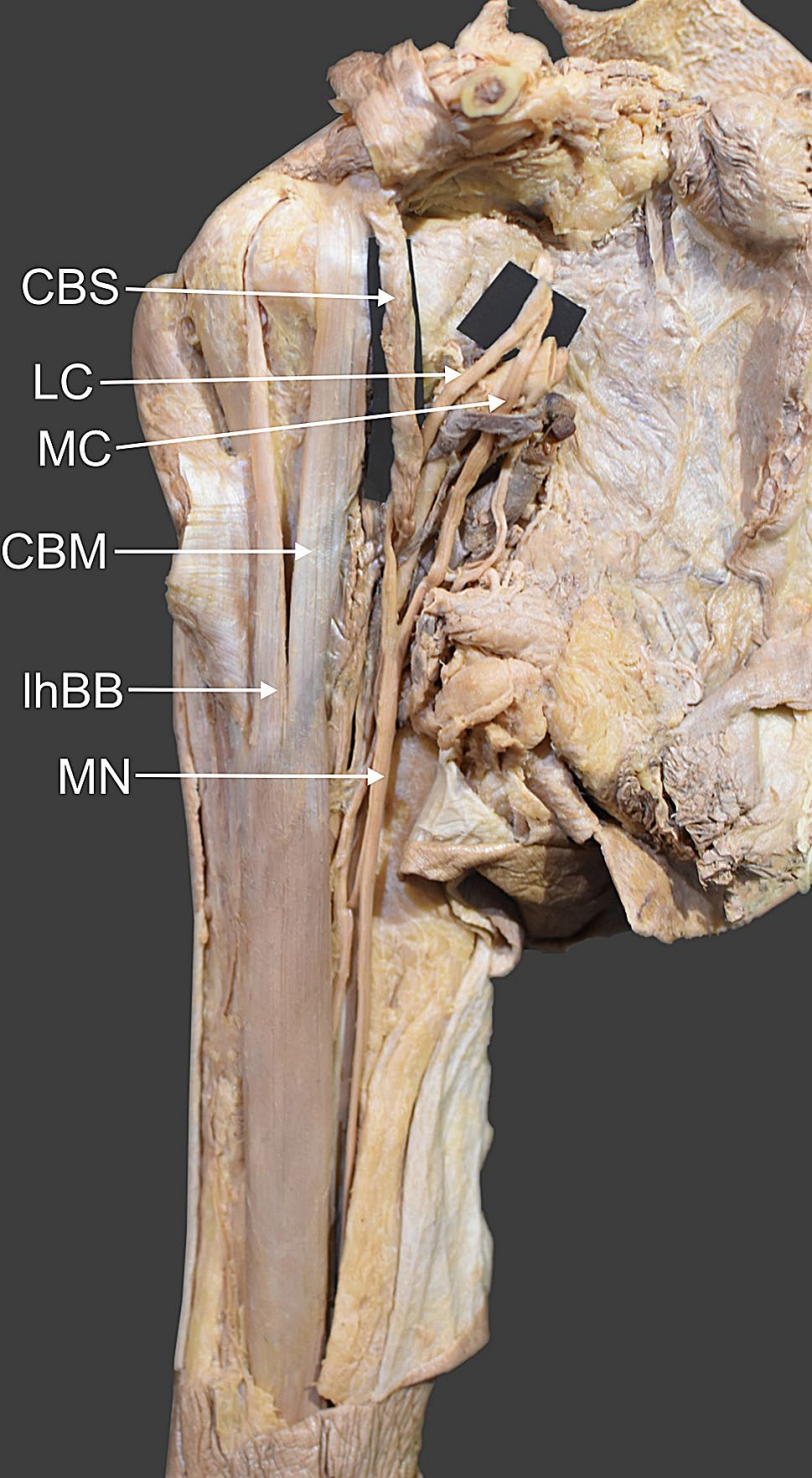
Coracobrachialis superior muscle. *CBS* coracobrachialis superior muscle *LC* lateral root of the median nerve *MC* medial root of the median nerve *CBM* coracobrachialis muscle *lhBB* long head of the biceps brachii *MN* median nerve

**Fig. 2:**
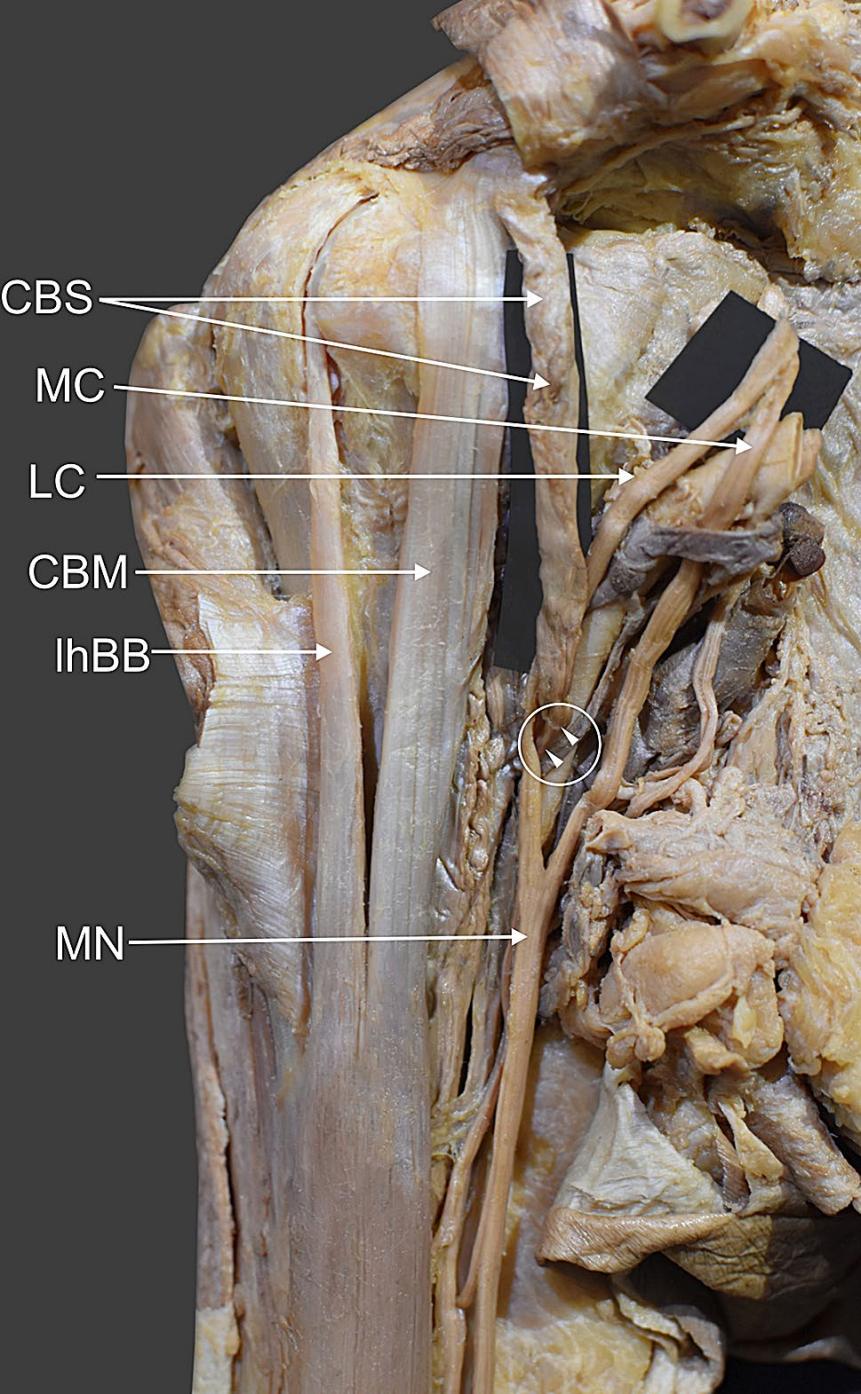
Coracobrachialis superior muscle. *CBS* coracobrachialis superior muscle *LC* lateral root of the median nerve *MC* medial root of the median nerve *CBM* coracobrachialis muscle *lhBB* long head of the biceps brachii *MN* median nerve. The white arrowheads shows insertion of the coracobrachialis superior muscle

The right upper limb was dissected as described previously [15]. Dissection revealed a morphological variant of the CBM and an unrecognized structure (Figs. 1, 2). The CBM corresponded to the classical description and originated from the apex of the coracoid process together with the short head of the biceps brachii. The muscle belly origin was 10.98 mm wide and 5.14 mm thick. The length of the muscle belly was 134.31 mm. The distal attachment was on the 1/3 of the humerus.

The CBS had attachment on the coracoid process above the common origin of the CBM and short head of the biceps brachii (common junction had a width 9.44 and 2.45 mm thick). At the proximal attachment site, it was 4.43 mm wide and 1.76 mm thick. The length was 69.73 mm. During its course it crossed with the lateral root of the median nerve. At the crossing with the lateral root of the CBS, it was 4.25 mm wide and 2.38 mm thick. The lateral root of the median nerve at the junction was 2.68 mm wide and 1.02 mm thick. Distally, its fibers connected to the CBM and had an attachment to the superior part of the shaft of the humerus. At the distal attachment site, it was 6.89 mm and 2.13 mm thick—Table 1.

**Table 1:** Morphometric measurements of individual parts of the coracobrachialis superior muscle

	Proximal attachment	Distal attachments
Width	4.43 mm	6.89 mm
Thickness	1.76 mm	2.13 mm

Detailed morphometric measurements were taken. After photographic documentation, the CBM was carefully dissected to minimize any errors in measurement. The measurements was performed using an electronic caliper (Mitutoyo Corporation, Kawasaki-shi, Kanagawa, Japan). Each measurement was carried out twice with an accuracy of up to 0.1 mm.

## Discussion

The CBM is characterized by a much greater morphological variability than previously thought [14–16]. Despite being quite small and inconspicuous, and mistakenly thought to have lacked any important function, this muscle is also of great clinical significance. The CBM is positioned on the medial side of the upper arm; it is believed to be the sole remaining muscle of the medial group of the upper arm, which is believed to have disappeared phylogenetically upon adopting a fully upright posture [19]. Wood [19] carried out a study in which assessed collocation of the CBM among various animals. For example, in the bonnet monkey, there are two portions of the CBM which create a common junction with the short head of the biceps brachii. The first part (short) is directly attached to the coracoid process by its fleshy fibres. Its insertion is located on the neck of the humerus above the tendinous part of the teres major and latissimus dorsi muscles (as in the human variety). In turn, the distal attachment of the second part is located on the inner surface of humerus between triceps brachii and brachialis anticus muscles [19].

In the hedgehog the CBM is represented by one part arising from the coracoid process. It inserts into the middle of the humerus. Meckel also examined the CBM in the hedgehog, but described it as a double muscle, which lower tendinous insertion is prolonged downwards.

Another examined group was the three-thoed slot. A single CBM is slender. Its distal attachment is located on the middle of the humerus, just below the teres major and latissimus dorsi muscles [19].

A single CBM also occurs among the armadillo. It is represented by long variant inserted upon the supra-condyloid arch or foramen just above the inner condyle [19].

The CBM in the dog and cat is characterized by short variety arising from a diminutive coracoid process. Its insertion is located in the region of the neck of the humerus above the latissimus dorsi and teres major muscles [19].

The guinea-pig and rabbit have been also examined. In these animals the CBM is single and its distal attachment is located just below the latissimus dorsi and teres major muscles.

The squirrel was another group studied by Wood. The CBM among this animal, is characterized by insertion located on the end of the humerus. Moreover, Meckel et al., found that the CBM is single, very long and strong [19].

Wood examined the CBM among the kangaroo rat. It turned out, that it is a short, very small variant which distal attachment is located above the tendinous part of the teres major and latissimus dorsi muscles [19].

In the echidna hytrix one part of the CBM is really large. Its proximal attachment is located on the coracoid process, and creates a common junction with the short head of the biceps brachii muscle. In turn, its distal attachment is located on the prominent condyle. There is also a distinct part originated to the coracoid process, but deeper, and inserted into the lower part of the transversely prominent inner or ulnar tuberosity of the humerus [19].

In the Ornithorhynchus paradoxux the CBM has a long part which is smaller than in the echidna. Its upper part is created by fibres arising from the coracoid head of the double-headed flexor radii, or short head of the biceps brachii. The second part of the CBM is attached below the brachial vessels and nerves upon the supra-condyloid arch or foramen, close above the epitrochlea. Both part may be connected with biceps brachii [19].

In both echidna and ornithorhynchus there is something like the short muscle, called by Meckel the coracobrachialis superior. Its proximal attachment is located on the coracoid process, deeper than the biceps brachii muscle or long portion of the CBM. It inserts into the ulnar tuberosity of the humerus, above the teres major and latissimus dorsi muscles [19].

It is worth mentioning, that Wood suggested that such a variable arrangement seems to be an effect of the wants and habits of animals. For example, animals using the fore-limbs for digging, climbing, swimming or distinot prehension, have a larger and better developed structure of the CBM [19].

Testut [17] describes that coracobrachialis muscle may not be present in some animal species. He mentions in his book that prof. Vrolik did not find this muscle in chimpanzees, however, Testut found and morphologically resembled a muscle found in humans [17]. In some animal species, in addition, that the musculocutaneous nerve never perforates the CBM, especially those which do not have the anterior compartment of muscle, such species are: chimpanzee, the orang, some Cercopithecus, the Chinese bonnet, the fox, the Solipeds, the Ruminants, the pig, the wolf [17].

The recorded morphological variations in the CBM concern mainly its relationship with the musculocutaneous nerve. Recently, Szewczyk et al. [16] distinguished two types of nerve course in relation to CBM morphology: one type piercing the belly of the muscle (one head) while the other passing between the heads of the CBM.

Further variations concern the morphology of CBM. The CBM consists of two heads; a superficial (anterior) head and a deep (posterior) head [6, 13]. The superficial head originates from the medial border of the tendon of the short head of the biceps brachii, while the deep head originates from the coracoid process of the scapula and the adjoining part of the lateral border of the tendon of the short head of the biceps brachii [6]. The deep layer of the CBM can originate from the insertion of the pectoralis major [13]. Mori [13] divides the CBM thus: Type A—The belly of the muscle is completely separated into its component superficial and deep layers (observed in eight arms), Type Bb—The belly of the muscle is incompletely separated into its component parts (four arms) and Type C—The belly shows no signs of dissociation into superficial or deep layers (38 arms).

Interestingly, one case has been reported of a three-headed CBM, characterized by a single superficial head and a deep head split into two [6]. In contrast, Ilayperuma et al. [11] do not report any such morphological variations in the CBM proximal attachment: they describe three possible proximal attachments for a single belly relative to the tendon of the biceps brachii [11], these being lateral to the tendon, medial to the tendon and deep to the origin of the tendon of the biceps brachii. In addition, Olewnik et al. [15] report the occurrence of a four-headed CBM with split coracoid process. The first two heads were arranged in layers and had a proximal attachment located on the *accessory apex*, while the third head, together with the short head of the biceps brachii, was on the coracoid process, and the fourth head was under the third and the short head of the biceps brachii (deep layer) [15].

Szewczyk et al. [16] proposed a separate classification for both the proximal and distal attachment: a threefold CBM classification (Types I–III) for the proximal attachment. The most common type was Type I (49.5%), which was characterized by a single belly with the origin located on the coracoid process, medially and posteriorly to the tendon of the short head of the biceps brachii. The second most common type was Type II (42.6%), which was characterized by the occurrence of two bellies. This type was divided into two subtypes: A and B. In Type IIA, the first head originates from the coracoid process posterior to the tendon of the short head of the biceps brachii and the second head originates from the short head of the biceps brachii. In Type IIB, both heads originate from the coracoid process; however, the superficial head fuses with the origin of the short head of the biceps brachii, while the deep head is directly originated. The rarest type was Type III (7.9%), which was characterized by a three-headed CBM; two heads (superficial and deep) originate from the coracoid process, whereas, the third arises from the short head of the biceps brachii.

Although the classification by Szewczyk et al. [16] appears quite comprehensive, some very rare varieties of CBM have also been recorded, such as the CBL or CBS (also called coracobrachialis brevis) as well as the coracocapsularis or additional bands originating from the CBM [2, 4, 12, 19]. Calori [3] found coracobrachialis minor (coracobrachialis brevis) muscle twice, one in female and one in man. Both cases occurred in the right upper limb. The proximal attachment was located on the base and tip of the coracoid process. The belly course downwards over the gleno-humeral joint and covered the subscapularis muscle. There was a small connection between the coracobrachialis minor and subscapularis muscle. The distal attachment was located on the lesser tuberosity. Cruveilher [4] found additional band fused with the tendon of latissimus dorsi. Wood [19] found and described interesting cases; the first was a small muscle, arising from the coracoid process, beneath the ordinary CBM and inserted into the neck of the humerus, below the insertion of the subscapularis. In turn the second very rare case described by Wood [19] was represented by a small slip from the fascia over the subscapularis tendon, beneath the coracoid process, passing down to the fascia derived from the tendon of the latissimus dorsi, covering the long head of the triceps brachii.

Wood [19], describes a very rare variant, the coracocapsularis muscle, which originates from the coracoid process and inserts into the shoulder capsule. Macallister [12], Theile [18], Testut [17], LeDouble [5] and Gruber [10] also described this muscle. Confusion often occurs when a variant has too many names. For example, Theile [18] and Gruber [10] named the tensor capsulae, later Gruber [10] called the same variant, the depressor which is synonomus with retinaculum musculare tendinis subscapularis majoris and capsularis humero-scapularis superior and finally, Macalister [12] named the same muscle coracocapsularis muscle like Wood [19].

The CBS was described first time by Cruveilher [4]. Its proximal attachment was to the base of the coracoid process, and the distal attachment below the lesser tuberosity of the humerus cover by the subscapularis muscle. A very interesting case was described by Beattie [1], where a coracobrachialis brevis muscle was observed on both sides. On the left side, the muscle arose by a fleshy belly from the anterior surface of the coracoid process, at the junction of the horizontal and vertical portions, about 3 cm from the tip of the coracoid. It passed downwards and outwards, with a slight upward concavity, over the anterior surface of the subscapularis. It was covered anteriorly by the pectoralis minor, CBM, and the short head of the biceps; it inserted into the medial bicipital ridge, about 1 cm below the lesser tubercle, and a fascial expansion continued into the tendon of insertion of the latissimus dorsi [1].

The CBL muscle is also very rare. The CBL might attach to the humerus, medial epicondyle or a fibrous band of the medial intramuscular septum, i.e. Struther’s ligament [2, 8, 9, 14, 19], it may also inserted to the tendinous part of the latissimus dorsi [4]. Olewnik et al. [14] described a very unusual type of CBL which had a distal attachment on an olecranon. In addition, the CBL could place compression on the musculocutaneous, ulnar and median nerve [14].

Thaile has described the CBM as a part with a deltoid muscle. Garbelotti et al. [7] report an interesting case of a CBM muscle with two heads. The lateral head followed its normal course until insertion into the middle third of the humerus, while the accessory head involved the lateral cord of the brachial plexus at 11.64 cm from the coracoid process before insertion into the intermuscular septum in the proximal third of the humerus.

Both of the above are very similar to the present case report. Their findings raise the question of whether the muscle described in the present study is a CBS or a rare variety of CBM. The close relationship of this muscle with the CBM could suggest that it is the latter; however, no such type was observed by Szewczyk et al. [16] when preparing a classification based on 101 limbs. Its classification is also based on the number of bellies, but they are rather layered. On the other hand, its course and attachments and relation to the lateral cord of the median nerve suggest that it is a CBS.

## Conclusion

The coracobrachialis muscle is characterized by great morphological variability. Two important variations are the coracocapsularis and coracobrachialis superior muscle.

## Data Availability

Please contact authors for data requests (Łukasz Olewnik PhD - email address: lukasz.olewnik@umed.lodz.pl).
